# Evaluation of the Vascular Endothelial Growth Factor and Smooth Muscle Actin Expression With Microvessel Density and Morphometric Analysis of Endometrial Vessels in Patients With Abnormal Uterine Bleeding

**DOI:** 10.7759/cureus.64125

**Published:** 2024-07-09

**Authors:** Kodavali Mahabharathi, Tanya Sharma, Soma Mukherjee, Deepti Joshi, Neelkamal Kapoor

**Affiliations:** 1 Pathology and Laboratory Medicine, All India Institute of Medical Sciences, Bhopal, Bhopal, IND; 2 Obstetrics and Gynaecology, All India Institute of Medical Sciences, Bhopal, Bhopal, IND

**Keywords:** angiogenesis, smooth-muscle actin, vascular endothelial growth factor, microvessel density, morphometry, abnormal uterine bleeding

## Abstract

Background: Abnormal uterine bleeding (AUB) that occurs in a structurally normal uterus with regular menstrual cycles and without other identifiable etiology is often caused by a primary endometrial disorder (AUB-E). Altered vascular morphological changes and expression of markers of angiogenesis have been implicated as an underlying cause in these cases.

Objectives: The study was conducted to investigate the expression of vascular endothelial growth factor (VEGF) and smooth muscle actin-alpha (SMA-α), and to perform microvessel density (MVD), and morphometric evaluation of endometrial vessels in patients with AUB-E.

Material and methods: Endometrial biopsies and hysterectomy specimens of 40 patients clinically diagnosed with AUB-E were included in the study with 40 age-matched controls. Immunohistochemistry (IHC) with VEGF and SMA-α was performed, and the expression and staining pattern was recorded as the number of positive vessels per 10 high power fields and intensity scores. Morphometric analysis was performed on CD34 stained sections using Leica Application Suite, version 4.4.0 software (Leica Microsystems, Wetzlar, Germany). MVD was calculated by the vascular hotspot method.

Results: A statistically significant increase in VEGF vessel count (p-value<0.001) and a decline in SMA-α expression (p-value=0.23) was seen in cases as compared to the control group. There was a statistically significant increase in microvessel caliber (p-value=0.01) and MVD (p-value <0.001) in cases as compared to controls.

Conclusion: These findings support aberrant vascular proliferation and impaired vessel maturation, contributing to the pathology of AUB-E. Alterations in angiogenesis in these patients reveal potential therapeutic targets for AUB.

## Introduction

Abnormal uterine bleeding (AUB), defined by deviations in the frequency, regularity, duration, or volume of menstrual flow in non-pregnant women, continues to challenge women’s health despite advancements in diagnostics and treatments. Affecting up to one-third of women during their reproductive years, AUB can severely impact physical, social, and psychological well-being. The International Federation of Gynecology and Obstetrics (FIGO) Menstrual Disorders Group (FMDG) has categorized the causes of AUB under the acronym ‘PALM-COEIN,’ representing polyp (AUB-P), adenomyosis (AUB-A), leiomyoma (AUB-L), malignancy (AUB-M), coagulopathy (AUB-C), ovulatory disorders (AUB-O), endometrial (AUB-E), iatrogenic (AUB-I), and not otherwise classified (AUB-N) [1-3].

The shedding and repair of the endometrial functionalis layer are influenced by key factors such as angiogenesis, hypoxia, and hemostasis [4]. Angiogenesis is crucial for the repair and regrowth of the endometrium following menses and is regulated by various growth factors, including epidermal growth factor (EGF), transforming growth factor (TGF), platelet-derived growth factor (PDGF), tumor necrosis factor (TNF), vascular endothelial growth factor (VEGF), and hypoxia-inducible factor-1α (HIF-1α) [5-10].

Women with regular, cyclic menses who experience heavy menstrual bleeding (HMB) or intermenstrual bleeding without other identifiable cause often have a primary disorder of the endometrium (AUB-E). Anomalous endometrial angiogenesis and vessel maturation have been implicated in AUB-E. Although several studies have investigated proangiogenic and antiangiogenic factors in these patients, further research and validation are needed. Exploring the alterations in angiogenesis in these patients could reveal new treatment targets for AUB and offer valuable insights for potential diagnostic and therapeutic strategies.

The study was conducted to investigate the expression of VEGF and smooth muscle actin-alpha (SMA-α) and to perform microvessel density (MVD), and morphometric evaluation of endometrial vessels in patients with AUB-E.

## Materials and methods

A cross-sectional prospective study was conducted in the Department of Pathology and Lab Medicine at a tertiary care hospital in Central India between January 2019 and June 2020. Approval was obtained from the Institutional Human Ethics Committee. The study included endometrial biopsies and hysterectomy specimens from patients presenting with chronic AUB with no clinically or radiologically identifiable cause. Patients with known pathological causes for uterine bleeding, such as gynecological malignancy, endometritis, adenomyosis, fibroids, or systemic causes of menorrhagia (e.g., thrombocytopenia, sepsis), as well as those with fragmented or inadequate endometrial biopsies, were excluded from the study.

The control group comprised age-matched women with primary or secondary infertility, prolapsed uterus, cervical lesions, or those undergoing tubal ligation with regular menstrual cycles. Written informed consent was obtained from all study participants. Demographic and clinical details were recorded as per the study proforma.

Immunohistochemistry (IHC)

Representative blocks from each case were selected for IHC. Sections were taken on poly L-lysin-coated positively charged hydrophobic slides. IHC was performed using the VENTANA Benchmark ULTRA automated immunohistochemical slide staining system (Roche Diagnostics, Tucson, USA) according to the manufacturer's protocol. The primary antibodies used were:

· CD34 (CD34 QB end 10, Mouse monoclonal antibody; 3 mL predilute Ready To Use (RTU), PathnSitu, Livermore, CA-94551 USA)

· SMA-α (actin-1A4, Mouse monoclonal antibody, 3 mL predilute RTU; PathnSitu, Livermore, CA-94551 USA)

· VEGF (RBT-VEGF, Rabbit monoclonal antibody, 3 mL predilute; Bio SB, CA 93117, USA)

VEGF and SMA-α expressions

The staining of blood vessels was graded as follows:

· 0: No detectable staining

· 1: Faint staining

· 2: Weak staining

· 3: Strong staining

Scores of 1, 2, and 3 were considered positive. The number of blood vessels staining positive for VEGF and SMA-α was counted in 10 high-power fields (HPF) and expressed as the number of positive blood vessels per HPF. Blood vessels and myometrial smooth muscles were used as internal controls.

MVD and microvessel caliber (MVC)

Histopathological sections stained with CD34 were used to visualize microvessels. MVD was calculated using the vascular hotspot method. Five areas with the highest density of microvessels (hotspots) in the endometrium at low magnification were selected. Vessels were counted in 10 fields in these areas at high magnification (X400). The mean number of vessels in an HPF at X400 represented the MVD of the tissue. Images from five HPFs (X400) in the hotspot area were captured for each sample using a Leica Microscope (Model Leica DM 750; Leica Microsystems, Wetzlar, Germany) and an Olympus microscope BX43F (Olympus Corporation, Shinjuku, Tokyo, Japan).

Arterioles, capillaries, and venules were analyzed for cytomorphometric analysis of mean vascular caliber. Arterioles were identified by the presence of one to two layers of smooth muscle cells, capillaries by a single layer of endothelial cells without smooth muscle cells, and venules by larger vessels with endothelial lining and occasional pericytes. Morphometric analysis was performed on CD34 immunostained sections. Twenty-five circular or elliptical microvessels from high MVD areas were selected to calculate the mean MVC using Leica Application Suite version 4.4.0 (Build: 454) software (Leica Microsystems, Wetzlar, Germany). For elliptical vessels, the minor axis was considered the caliber. MVC was measured in micrometers (µm).

Statistical analysis

Data were analyzed using the Statistical Package for the Social Sciences (IBM SPSS Statistics for Windows, IBM Corp., Version 23.0, Armonk, NY). Descriptive statistics included frequencies, proportions, mean ± standard deviations, and median (interquartile range). Categorical variables were reported as proportions and continuous data as mean ±2 standard deviations. Statistical differences between categorical variables were assessed using Fisher's exact test, and for continuous variables, the student's 't' test (for normally distributed variables) and the Mann-Whitney U test (for non-normally distributed variables). P-values less than 0.05 were considered statistically significant.

## Results

A total of 40 endometrial biopsies from cases presenting with AUB fulfilling the inclusion criteria were analyzed. None of the cases had a history of drug or hormonal intake or intrauterine contraception for at least three months prior to histopathological sampling. Physical and radiological examinations were normal in all cases. Cervical pap smears were negative for intraepithelial lesions or malignancy in all cases. Patients' ages ranged from 32 to 55 years, with most cases in the 35-45 years age group. Forty age-matched controls were included in the study.

Superficial lining epithelium was identified and documented in all cases to ensure analysis of the functional layer of the endometrium. Among the cases, 26 (65%) had secretory endometrium, 10 (25%) had proliferative endometrium, and four (10%) had disordered proliferative endometrium. In the control group, 10 (50%) had secretory endometrium, seven (35%) had proliferative endometrium, and three (15%) had disordered proliferative endometrium.

VEGF expression

**Figure 1:**
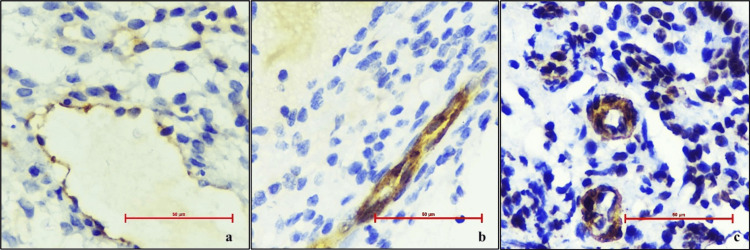
(a) Microphotograph showing vascular endothelial growth factor (VEGF) expression in cases with staining intensity score 1+; (b) Microphotograph showing VEGF expression in cases with staining intensity score 2+; (c) Microphotograph showing VEGF expression in cases with staining intensity score 3+ (400X, DAB chromogen).

**Figure 2:**
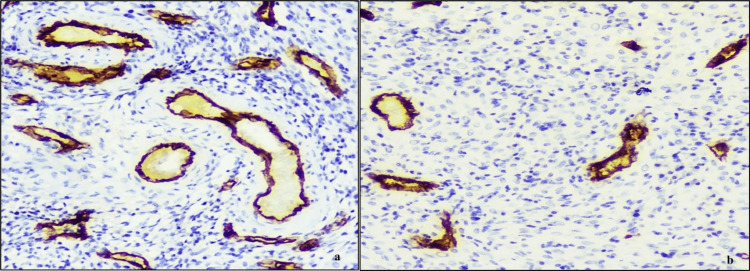
Microphotograph showing vascular endothelial growth factor expression in cases with higher mean vessel count/HPF in cases of AUB (a), compared to controls (b) (400X, DAB chromogen). HPF: high power fields; AUB: abnormal uterine bleeding

VEGF was expressed as mean positive vessel count/HPF (average of vessels counted in 10 HPF) and intensity score (1, 2, 3) for both cases and controls (Figures 1-2).

The mean VEGF positive vessel count per HPF was 31.4 in cases and 14.05 in controls, showing a significant increase in VEGF vessel count in cases (p<0.001) (Table 1). The mean intensity score was 1.55 in cases and 1.15 in controls (Table 2).

**Table 1:** Table depicting mean vascular caliber, microvessel density, vascular endothelial growth factor (VEGF), and smooth muscle actin-alpha (SMA-α) staining among cases of abnormal uterine bleeding and controls.

Parameters	Cases (n=40)	Controls (n=40)	p-value
	Mean±SD	Mean±SD	
Mean vascular caliber (µm)	21.02±9.2	16.6±3.8	0.01
Microvessel density	22.7±3.9	19.7±1.9	<0.001
VEGF (vessel count)	31±14	14±8	<0.001
SMA (vessel count)	32±14	38.4±13	0.23

**Table 2:** Vascular endothelial growth factor (VEGF) and smooth muscle actin-alpha (SMA-α) staining intensity score analysis of cases of abnormal uterine bleeding and controls.

Parameters		Group				p-value
		Cases		Controls		
		Count	n %	Count	n %	
VEGF intensity score	0	0	0	0	0	0.1
	1+	19	47.5	30	75.0	
	2+	20	50.0	10	25.0	
	3+	1	2.5	0	0	
SMA intensity score	0	0	0	0	0	1
	1+	2	5.0	2	5.0	
	2+	27	67.5	28	70.0	
	3+	11	27.5	10	25.0	

VEGF expression also showed a significant increase in the proliferative (p=0.008) and secretory phases (p=0.001) among cases and controls (Table 3).

**Table 3:** Table depicting mean vascular caliber, microvessel density, vascular endothelial growth factor (VEGF), and smooth muscle actin-alpha (SMA-α) staining among cases of abnormal uterine bleeding in the secretory phase versus the proliferative phase.

Parameters	Secretory phase		p-value	Proliferative phase		p-value
	Controls	Cases		Controls	Cases	
	Mean±SD	Mean±SD		Mean±SD	Mean±SD	
Mean vascular caliber	17.7±2.9	20.4±7.4	0.287	15.4±4.4	22.1±12.2	0.112
Microvessel density	20.1±2.0	22.9±3.1	0.011	19.3±1.8	22.4±5.2	0.088
VEGF (vessel count)	13±8	30±10	0.001	16±8	34±18	0.008
SMA (vessel count)	40±11	32±14	0.118	33±15	32±15	0.862

SMA-α expression

**Figure 3:**
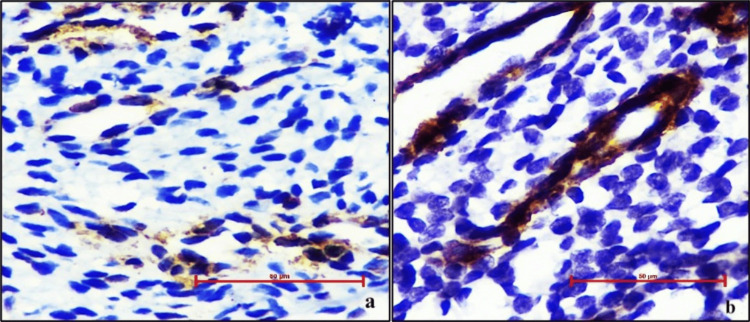
(a) Microphotograph showing smooth muscle actin-alpha (SMA-α) expression in a case of abnormal uterine bleeding (AUB) with staining intensity score 1+; (b) Microphotograph showing SMA-α expression in controls with staining intensity score 2+ (400X, DAB chromogen).

SMA-α was expressed as the mean positive vessel count per HPF and intensity score for both cases and controls. The mean positive vessel count per HPF was 32.35 in cases and 38.4 in controls. A decline in SMA-α expression was observed among cases, however, no statistically significant difference was found compared to controls (p=0.23). The mean intensity score was 2.15 in cases and 2.35 in controls (Figure 3, Table 3).

No significant difference was observed in SMA-α mean vessel count in the proliferative and secretory phases among cases and controls (Table 2).

MVC

The mean MVC was 21.03 µm in cases as compared to 16.6 µm among the controls with a statistically significant increase in MVC in the cases as compared to controls (p-value=0.01) (Table 1). No statistically significant difference was found in the mean MVC in the proliferative and secretory phases among cases and controls (Table 3).

MVD

**Figure 4:**
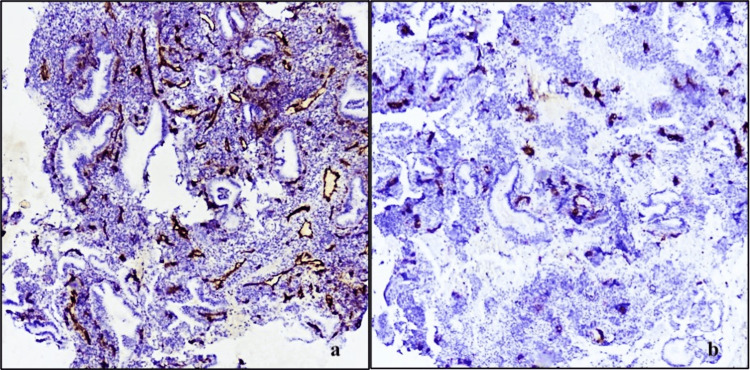
(a) Microphotograph showing hotspot for analysis of microvessel density in CD34 stained sections of cases (l00X, DAB chromogen); (b): Microphotograph showing hotspot for analysis of microvessel density in CD34 stained sections of controls (l00X, DAB chromogen).

The mean MVD was 22.7 in the cases as compared to 19.7 among the controls with a statistically significant increase in MVD of cases as compared to controls with a p-value<0.001 (Figure 4). MVD showed a statistically significant increase in microvessels in the secretory phase (p=0.011) and the proliferative phase (p=0.088) among the cases and controls (Table 3).

## Discussion

AUB that occurs in a structurally normal uterus with regular menstrual cycles and without other identifiable etiology is likely to have an underlying endometrial cause (AUB-E). Despite advancements in diagnostics and treatments, the role of markers involved in endometrial function in menstruation and its disorders remains a focus of ongoing scientific research. Recent studies suggest that altered vascular morphological changes and the expression of angiogenesis markers may underlie these cases. In the present study, we observed a significant increase in VEGF-positive vessel count, MVD, and mean vascular caliber in cases of AUB-E. Additionally, we observed a decline in SMA-positive blood vessels in these cases. These findings indicate aberrant vascular proliferation and impaired vessel maturation, contributing to the pathology of AUB-E.

VEGF expression has been previously studied to understand its role in abnormal angiogenesis in women with HMB. Mints et al. evaluated VEGF expression in endometrial biopsies of 24 patients with menorrhagia and 18 women with regular menstrual bleeding, finding a statistically significant increase in VEGF-positive vessels in menorrhagia patients (p=0.001) [11]. Another study was conducted by Zhang et al. to depict the function of matrix metalloproteinase, VEGF, and microvascular density in anovulatory dysfunctional uterine bleeding (DUB) patients. They studied 60 anovulatory DUBs and controls. They concluded that increased expression of matrix metalloproteinase, VEGF, and high microvascular density in endometrial hyperplasia may play a key role in the pathogenesis of the anovulatory DUB [12].

Mehra et al. conducted a study to determine the correlation between the VEGF with endothelial cell proliferation markers like CD34 and proliferating cell nuclear antigen. They graded the staining intensity of VEGF among menorrhagic and normal women. The VEGF staining score was done in the layers of endometrium stratum basalis and stratum functionalis, endometrial vessels, epithelium of endometrial glands, and endometrium stroma. The scoring was given based on the degree of staining and intensity of staining. These scores were graded according to the staining intensity. They found a statistically significant increased endothelial cell proliferation during the late secretory phase in the menorrhagia patients compared to women with normal menstruation and also in the premenstrual phase (p≤0.001) [13]. In the present study, we analyzed the intensity of VEGF expression among the two groups. Although the mean intensity score was more in cases, compared to controls, no statistically significant difference was observed.

Few studies have also shown an association of VEGF-mediated angiogenesis with the development of adenomyosis and endometriosis [14,15]. VEGF has been put forward as a potential diagnostic and therapeutic marker. Studies have been conducted to study the efficacy of inhibitors of VEGF/VEGF receptors (VEGFRs), like gonadotropin hormone-releasing hormone (GnRH) agonists or anti-VEGF/VEGFR agents in various endometrial pathologies including AUB, endometriosis and malignancies [16-20].

Abberton et al. in 1996 conducted a study to compare the staining pattern of SMA-α in the endometrium of women with AUB and without menorrhagia and also in the women before perimenopause and during perimenopause. They collected 108 endometrial samples and grouped them into perimenopausal menorrhagic women, perimenopausal control women, and non-perimenopausal menorrhagic women, non-perimenopausal control women. They determined that a statistically significant less number of SMA-α stained vessels were seen in non-perimenopausal menorrhagic women (p=0.04) as compared to non-perimenopausal control, and also staining score was statistically significantly increased in the cases of perimenopausal menorrhagic women than perimenopausal control women [21].

Abberton et al. in 1999 conducted another study to determine the alteration in the endometrial vascular smooth muscle spiral arterioles growth and development which contributes to the menorrhagia. They performed SMA-α and myosin heavy chain on the endometrial samples with 64 samples of 24 menorrhagia and 40 controls. They reported a statistically significant reduction in SMA-α expression in the spiral arterioles in women with AUB than controls (p<0.05) [22].

Andersson et al. in 2014 conducted a study using a confocal microscope to evaluate the pericyte coverage which was immunohistochemically stained with SMA-α in 10 women with HMB of endometrial origin and 17 controls. They found that the number of vessels stained with SMA in the proliferative phase was significantly lower in the women with HMB of endometrial origin than in controls (p=0.005) However, there was no statistical difference observed in the secretory and proliferative phases in the women with HMB. They reported that VEGF-stained vessels showed a negative correlation with the microvessels covered with pericytes [23]. An increase in MVC and reduced pericyte coverage could be a sign of maturation, though when pericyte coverage is absent and wall gaps and defects are increased, this can lead to fragile and leaking microvessels, thereby causing AUB.

Mints M et al. in 2005 performed a study to evaluate the MVD in patients with idiopathic menorrhagia. They found no statistically significant difference in the MVD between menorrhagia patients and healthy controls. However, they found that in menorrhagia patients, MVD was statistically significantly more in the secretory phase as compared to the proliferative phase. In the present study, we found a statistically significant increase in MVD in cases as compared to controls [24].

Mints M et al. in 2007 conducted a study to evaluate underlying abnormalities in the structure of blood vessels in the endometrium of women with idiopathic menorrhagia. They performed morphometrical analysis on CD34-stained blood vessels in the functionalis layer of endometrium in 24 cases and 18 controls. They found the diameter and circumference of endometrial blood vessels in the secretory phase to be significantly greater in women with menorrhagia as compared to controls with p-values of 0.001 and 0.0007, respectively [11].

Zhang et al. found that there was a statistically significant increase in MVD between the anovulatory DUB and healthy controls summarized [12]. In another study, Andersson et al. reported that MVD did not show any statistical difference between the women with HMB and controls (p>0.05) [23]. Makhija et al. in 2008 studied 500 endometrial specimens to evaluate alterations in blood vessels in various phases of the menstrual cycle, menstrual disturbances, and unexplained infertility. They included 437 cases and 63 controls in the study. They found that congestion and dilation of blood vessels were significantly higher in cases of DUB and concluded that endometrial angiogenesis correlates with menstrual disorders [25].

A recent review by Middelkoop et al. highlighted a significant increase in VEGF-A and its receptors, the angiopoietin-1 ratio, Tie-1, and differential expression of other pro- and antiangiogenic factors in patients with AUB-E. These findings overall support the presence of aberrant vascular maturation and impaired vessel integrity in AUB-E cases. Though MVD was found comparable among AUB-E cases and controls, a significant increase of MVD was reported in AUB-I. Although the MVD was found to be comparable among AUB-E cases and controls, a significant increase in MVD was reported in cases of AUB-I [5].

## Conclusions

The present study showed increased VEGF expression, corroborating its role in altered angiogenesis in AUB-E. Statistically significant increase in MVD and mean vascular caliber, and decline in SMA-α expression in AUB-E indicates altered blood flow regulation, reduced pericyte coverage, and impaired vascular maturation as underlying pathology. These findings reinforce the significance of vascular morphological alterations in menstrual bleeding disorders. In an era where targeted therapies against endometrial angiogenesis are emerging, this study provides valuable insights into potential diagnostic and therapeutic approaches for managing AUB.
